# Solitary Cecal Ulcer: A Case Report

**DOI:** 10.3389/fsurg.2022.819519

**Published:** 2022-04-01

**Authors:** Chun Qiang Li, An-Qi He, Gang Liu

**Affiliations:** Department of General Surgery, Tianjin Medical University General Hospital, Tianjin, China

**Keywords:** cecal ulcer, colonic neoplasms, inflammatory bowel disease, colorectal diseases, abdominal pain

## Abstract

**Background:**

Solitary cecal ulcer is a rare disease. Its etiology is unknown and there are no pathognomonic symptoms. There are rare reports mimicking carcinoma as seen in this case.

**Case Presentation:**

A 64 year-old woman presented with a history of intermittent right lower abdominal pain for 20 years and worsening for 1 year. Colonoscopy revealed an enormous cecal ulcer. The PET-CT showed increased metabolism of the lesion. She underwent a right hemicolectomy. Histopathological examination revealed chronic non-specific inflammation. A rare diagnosis of the solitary cecal ulcer was ultimately made.

**Conclusion:**

Solitary cecal ulcer is a rare, idiopathic entity. It mimics inflammatory bowel disease, malignancy, infection, etc. The comprehensive images of this case describe the characteristics of the disease.

## Introduction

Solitary cecal ulcer is rare. Its etiology is unknown, and there are no pathognomonic symptoms. Its clinical presentation is varied, but abdominal pain is common. The PET-CT or CT may suggest a carcinoma. The definitive diagnosis is made only on histopathological examinations. We describe comprehensive images including PET-CT, colonoscopy, specimen, and pathology. These images describe the characteristics of the disease. Meanwhile, we review pieces of literature on this rare disease.

## Case Presentation

**Figure 1:**
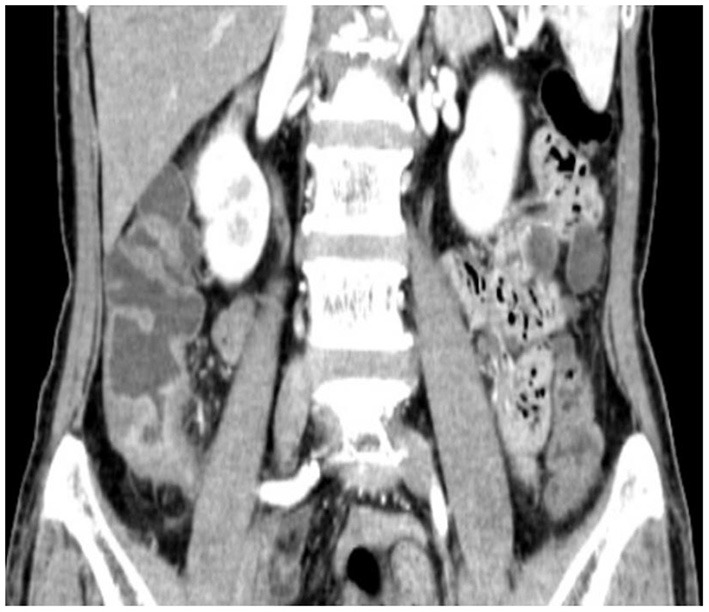
CT showed that the ileocecal intestinal wall was thickened.

**Figure 2:**
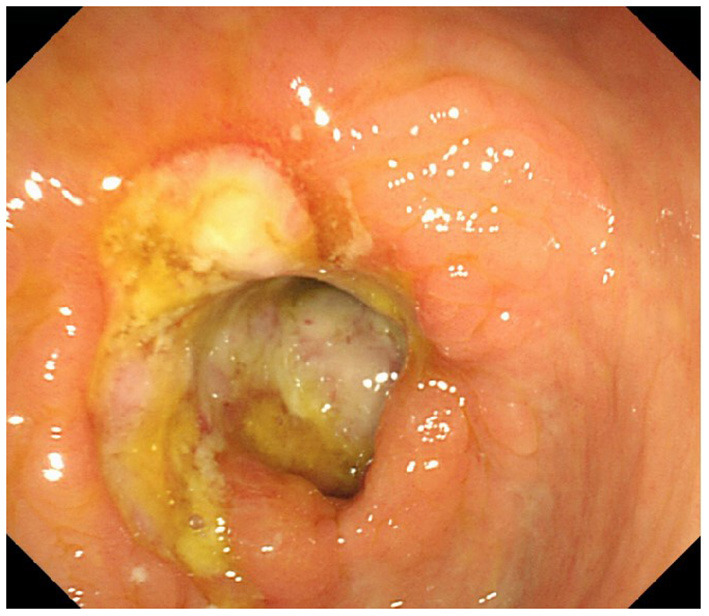
Colonoscopy revealed a cecal enormous ulcer.

**Figure 3:**
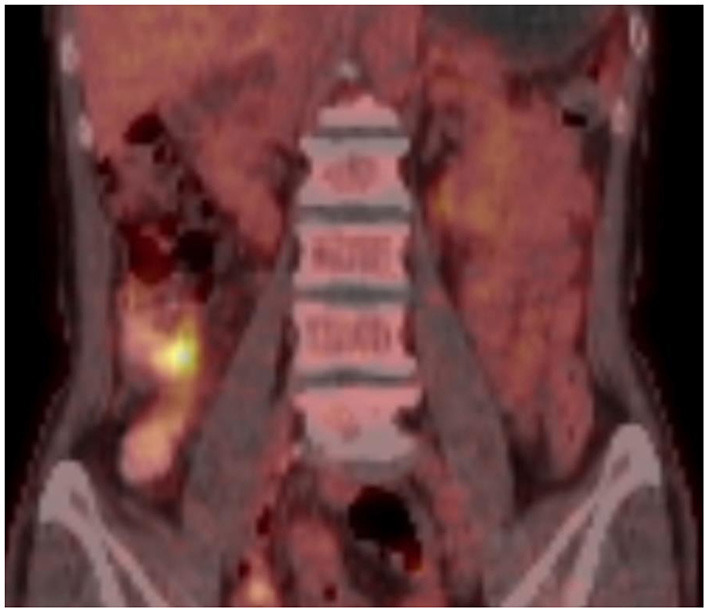
PET-CT showed the increased metabolism (SUVmax: 9.3) of the lesion.

**Figure 4:**
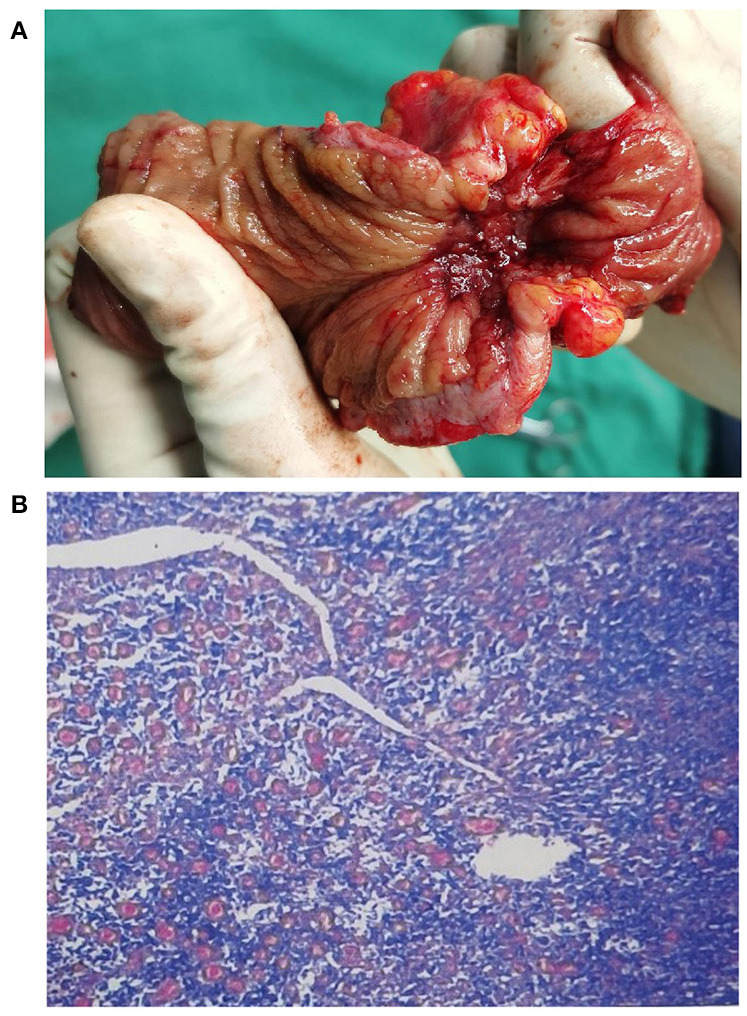
**(A)** An isolated, well-circumscribed ileocecal ulcer could be seen in the surgical specimen; **(B)** The post-operative pathological analysis revealed chronic non-specific inflammation (hematoxylin and eosin, original magnification 20 ×).

A 64 year-old woman presented to the outpatient department with a history of intermittent right lower abdominal pain for 20 years and worsening for 1 year with no weight loss. She was a non-smoker and had unremarkable medical and family medical histories. Recently, she took mesarazine intermittently without marked remission. A physical examination was notable for a palpable mass and tenderness on the right lower abdomen. The laboratory studies showed no abnormality, except for a c-reactive protein level of 2.58 mg per deciliter (reference range, <0.8). The CT imaging showed that the ileocecal intestinal wall was thickened (Figure 1). Colonoscopy revealed a cecal enormous ulcer (Figure 2). The pathology reminded of nothing except for non-specific inflammation, so a PET-CT was arranged and showed the increased metabolism (Figure 3, SUVmax: 9.3) of the lesion. She underwent a right hemicolectomy in case of a malignancy. An isolated, well-circumscribed ileocecal ulcer could be seen with a size of about 2^*^2 cm (Figure 4A). The histopathological examination (Figure 4B) revealed chronic inflammation and significant fibrosis at the edges and bottom of the ulcer, lymphocyte hyperplasia, and several lymph nodes were benign. The mucous crypt was normal with no evidence of granulomas, cancer. After exclusion of other possible diagnoses, the rare diagnosis of the solitary cecal ulcer was ultimately made. The patient remained well a year after surgery.

## Discussion

Solitary ulcer in the colon is rare. Only a few cases in the literature describe the characteristics of solitary cecal ulcer. McCarthy and Beveridge (1) and Last and Lavery (2) reported a case of solitary cecal ulcer with hemorrhage separately. Yeom et al. (3) reported a case of multiple pyogenic liver abscesses caused by the micro-perforation of idiopathic cecal ulcer. González-Urquijo et al. (4) reported a case of a patient presenting with a solitary cecal ulcer associated with non-steroidal anti-inflammatory drugs (NSAIDs) intake, mimicking a malignant lesion, and a laparoscopy right colectomy was performed. In addition, conditions that mimic appendicitis (5) and coexists appendix carcinoid tumors (6) have been reported. Ong et al. (7) described the experience of 10 patients with benign caecal ulcers.

The signs and symptoms of solitary cecal ulcers are non-specific, and the disease has no pathognomonic signs, which leads to the fact that the solitary cecal ulcer is often misdiagnosed as acute appendicitis (8). The most common symptom is right lower abdominal pain, but other symptoms include melena, constipation, diarrhea, and weight loss (5, 8, 9). Diagnosis is difficult to make preoperatively and intraoperatively, and definitive diagnosis is usually obtained by surgical and/or colonoscopic histological biopsy (10, 11).

Solitary cecal ulcer has a small incidence. They can appear in every age group but have a slightly higher incidence in patients aged 40–60 years and are more common in women (12, 13). The etiology is unknown, and other than the leading cause being consumption of non-steroid anti-inflammatory drugs (9, 14, 15), there are others such as the Solitary Rectal Ulcer Syndrome, sterocoraceus ulcer, ischemic ulcer, or ulcers caused by infections, among other even less common causes (9, 13). These etiologies are most commonly found in the left colon and rectum, and NSAIDs ulcers are mainly found in the cecum and right colon.

The CT scan of a chronic solitary cecal ulcer is very similar to those of a cecal carcinoma, with the colonic wall thickening by edema in the sub-serous layer, striations in the pericolonic adipose tissue (9). This is consistent with the findings in our patient. This is a key element to explain our decision of the pathology reminded nothing except for non-specific inflammation, and the PET-CT showed characteristics mimicking carcinoma. Diagnosis relies on colonoscopy with a biopsy taken from the ulcer to rule out malignant lesions. In patients with a possible diagnosis of inflammatory bowel disease, random samples should be taken from the entire colon (9, 13).

The treatment should consist of the immediate discontinuation of NSAID therapy, a high fiber diet to avoid constipation, and conservative care at first (9). An operation should be considered when the symptoms persist, severe gastrointestinal bleeding secondary to cecal ulceration or a malignant lesion cannot be ruled out (7).

## Conclusion

In summary, solitary cecal ulcer is rare. Its etiology is unknown. It needs to be differentiated from malignant lesions, infection and inflammatory bowel diseases, etc. An operation should be considered when the symptoms persist, severe gastrointestinal bleeding secondary to cecal ulceration or a malignant lesion cannot be ruled out.

## Data Availability Statement

The original contributions presented in the study are included in the article/supplementary material, further inquiries can be directed to the corresponding author/s.

## Ethics Statement

Written informed consent was obtained from the individual(s) for the publication of any potentially identifiable images or data included in this article.

## Author Contributions

CL and A-QH wrote the manuscript and were assistants in surgery. GL was the chief operating surgeon. All authors contributed to the article and approved the submitted version.

## Conflict of Interest

The authors declare that the research was conducted in the absence of any commercial or financial relationships that could be construed as a potential conflict of interest.

## Publisher's Note

All claims expressed in this article are solely those of the authors and do not necessarily represent those of their affiliated organizations, or those of the publisher, the editors and the reviewers. Any product that may be evaluated in this article, or claim that may be made by its manufacturer, is not guaranteed or endorsed by the publisher.
